# Building Minimalist Models for Functionalized Metal Nanoparticles

**DOI:** 10.3389/fmolb.2019.00050

**Published:** 2019-07-02

**Authors:** Giorgia Brancolini, Valentina Tozzini

**Affiliations:** ^1^Istituto Nanoscienze–CNR-NANO S3, Modena, Italy; ^2^Istituto Nanoscienze–CNR and NEST-Scuola Normale Superiore, Pisa, Italy

**Keywords:** coarse grained models, molecular dynamics, brownian dynamics, multiscale simulations, gold nanocrystal, macromolecules aggregation

## The Landscape of Coarse Grained NP Models

Metal nanoparticles (NPs) have been recently proposed for an increasing number of applications in nano-medicine (Vlamidis and Voliani, 2018) and nanotechnology (Chen et al., 2015). For instance, gold NPs (Alex and Tiwari, 2015) allow covalent versatile functionalization *via* thiol chemistry (Hakkinen, 2012) with different biomolecules or functional groups to selectively favor interactions with proteins or other specific components of the cell milieu. In particular, thiol-protected gold NPs functionalized with phenyl groups, Au_25_${\text{L}}_{18}^{-}$ (L = S(CH_2_)_2_Ph) were considered capable of interfering with protein aggregation, and therefore viewed as possible therapeutic agents against degenerative diseases due to amyloid fibrils accumulation (Brancolini et al., 2014, 2018; Marcinko et al., 2017; Torsten et al., 2018). The optimization of the size and decoration of the NP for therapy can benefit from computer simulations exploring aggregation in different environmental conditions (relative concentration, temperature, ionic strength). However, such extremely large time and size scale simulations call for the use of super-atomistic representations (low resolution or coarse grained—CG—models) (Brancolini and Tozzini, 2019).

A number of CG models for proteins are available (Seo et al., 2012), even minimalist ones, i.e. with single-bead per amino-acid resolution and implicit solvent (Di Fenza et al., 2009; Tozzini, 2010; Trovato and Tozzini, 2012; Trovato et al., 2013). Conversely, for the NPs, available CG models are rather sparse and diverse. The presence of the gold core suggests treating it at the meso-scale as a single spheroidal object (Vàcha et al., 2014), but the roughness of the surface (Radic et al., 2015), and the specificity of the chemical decoration (Tavanti et al., 2015a; Cantarutti et al., 2017) have fundamental roles in the interaction with proteins and must be treated at a higher resolution (Brancolini et al., 2015; Tavanti et al., 2015b; Charchar et al., 2016; Cardellini et al., 2019). Particular attention must be paid to the representation of hydrophobic character of the chemical groups and to the presence of possible net charges, whose medium- and long-range character, respectively, is the determinant of the macroscopic aggregation properties of the system. Implicit solvent requires the use of accurate screened potentials to account for the ionic strength. Finally, for the NP model to be compatible with the protein counterpart, both resolution and parameterization of the force field (FF) should be well matched.

While these prescriptions are followed inprevious literature in given models (Radic et al., 2015; Charchar et al., 2016), here we outline a general strategy to build models for NPs including all of them. In our view (Brancolini et al., 2018) these should contain the following ingredients: (1) *Minimalism*, i.e., including the minimum possible amount of degrees of freedom (DoF), and implicit solvent (2) *Compatibility* with the protein models (3) *Transferability* to different sizes and chemical decorations. Clearly, each of these characteristics involves one or more among the following actions: (i) choice of the model structure/topology, (ii) choice of the functional forms for the interactions, (iii) optimization of parameterization. (ii) and (iii) are complex tasks which have been addressed using a large number of different methodologies (Bauer et al., 2017; Lin et al., 2018; Brancolini et al., submitted). Particularly effective are usually combinations of bottom up and top-down strategy (Leonarski et al., 2013; Mereghetti et al., 2016) including both atomistic simulations and experimental data (Trovato and Tozzini, 2014) from different sources (e.g., structural, or thermodynamic). Here we focus on a general strategy to address (i) (Brancolini et al., 2018).

## Rational Building of a Minimalist NP Model

**Figure 1:**
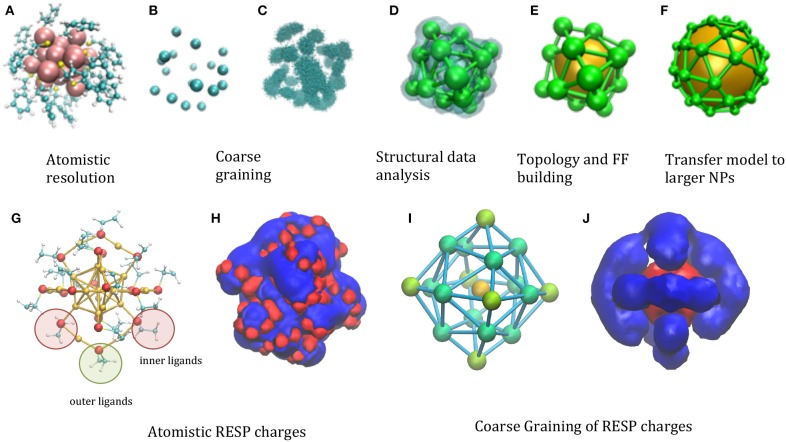
Illustration of the phases of the model building, taking as a test case the ${\text{Au}}_{25}{\left(\text{S}{\left({\text{CH}}_{2}\right)}_{2}\text{Ph}\right)}_{18}^{-}$. **(A)** Atomistic resolution, **(B)** coarse graining of functionalizing beads **(C)** analysis of the statistical structural data, clustering **(D)** evaluation of centroids of the space distribution, **(E)** topology and FF building **(F)** transfer of topology/structure to larger models. **(G)** Different symmetry of ligands are highlighted in the atomistic structure: 12 inner ligands (red circle) with sulfur bond to one core Au atom and one staple Au atom and 6 outer ligands (green circle) bound to two staple Au atoms. In principle, inner and outer beads can have different parameters, if needed to better balance the structure and electrostatics. **(H)** The electrostatic potential generated by the RESP atomistic charges using a representative configuration (UHBD isosurfaces drawn at +0.5 (red) and −0.5 (blue) kcal/e electrostatic potential). **(I)** The different types of CG beads reflecting the different symmetries of ligands are colored in different shades of green and assigned different charges [B2 = −0.230*e* (green), B3 = −0.128*e* (light green), the gold bead has charge B1 = +2.525*e;* the total net charge −1 is reproduced]. **(J)** CG electrostatic potential generated by the CG charges [UHBD isosurfaces drawn at +0.5 (red) and −0.5 (blue) kcal/e electrostatic potential].

The starting point is an atomistic structure of the functionalized NP (Figure 1A). The *minimalism* requirement suggests using a single large interacting center (“bead”) for the gold core, which is, in fact, a common feature to most of the NP models (Charchar et al., 2016; Shao and Hall, 2017). The chemical decoration is accounted for in several models by covering the central bead with smaller beads (Radic et al., 2015). The *compatibility* criterion can be satisfied choosing in specific ways the number and location of the decoration beads. For instance, when the functionalizing groups resemble in size and shape the side chains of amino-acids, this choice is rather straightforward: each of the functional group can be represented using the same representation of the protein amino-acids, i.e., 2–4 beads in MARTINI-like models (Seo et al., 2012), or a single bead for the minimalist models (Figure 1B). Remarkably, the model will include a number of DoF (Degrees of Freedom) proportional to the number of functional groups, i.e., will scale proportionally the surface of the NP, rather than to the volume.

An important point is how to choose the relative location of the decorating beads. Clearly, the thermal fluctuations of the group that they represent will determine the space distribution of the bead locations, which can be evaluated by means of atomistic simulations of small NPs (Maccari et al., 2014) (Figure 1C). The volume map build using this space distribution will form lobes, whose centroid and dispersion can be determined by clustering procedures (Arkhipov et al., 2006) (Figure 1D). This information can be used to build the starting location and topology of the model, and to parameterize the force field (FF) describing its internal dynamics (Figure 1E). Those parameters will then be transferred to larger NPs, once an average position of the functionalizing groups is determined, either from an atomistic model or from structural data (Figure 1F).

Distributing masses and effective charges among the beads is a non-trivial point. Considering masses, for instance, an obvious way would be to assign to each bead the sum of masses of their constituting elements. This, however, might not preserve the rotational inertia of the NP: in fact, being the total mass of the metal core concentrated in the center, it does not contribute, resulting in too small total rotational inertia. The problem can be solved by attributing larger masses to the peripheral beads. The proper balance of masses can be found by imposing that the total mass and the total rotational inertia correspond to that of the atomistic NP (Bauer et al., 2017).

The problem of charges is analogous: in this case an accurate charge distribution might be adjusted to reproduce the electrostatic potential, besides the net charge. The reference electrostatic potential can be generated from the RESP derived atomistic charges (Heaven et al., 2008), based on *ab initio* calculations (Figures 1G,H). Deriving the CG charges based on the atomistic components (Baker et al., 2001; Terakawa and Takada, 2014; McCullagh et al., 2016) results in effective charges depending on the bead type (gold or ligand) and symmetry (Figure 1I). The electrostatic potential generated by these can be compared with its atomistic counterpart, showing that the general shape of the iso-surfaces is preserved (Figure 1J): although of course the atomistic detail is lost, the CG model reproduce the global net prevalence of negative character (in blue), which however uncovers some positive areas (in red) for given directions, as in the atomistic case.

## Summary and Perspectives: The Next Steps

In our opinion, the presented strategy includes all the crucial elements of an optimal low resolution model: the choice of the *minimal* possible resolution, *compatibility* between different levels of resolution, a parameterization including the specific coating present on the NP by means of superficial higher resolution interacting sites. The effective charges could be further optimized by directly adopting a RESP procedure for their fitting. This task and the model validation at different concentrations and ionic strengths vs. the aggregation tendency are currently in due course (Brancolini et al., submitted). The following steps will be the use of the model in combination with proteins models at the same CG level (minimalist), to verify their effective capability of preventing the amyloids aggregation. Furthermore, the strategy here outlined is extensible to larger NPs and different functionalization, which opens the possibility of *in silico* optimization of the NPs size and chemistry for therapeutic use.

## Author Contributions

All authors listed have made a substantial, direct and intellectual contribution to the work, and approved it for publication.

### Conflict of Interest Statement

The authors declare that the research was conducted in the absence of any commercial or financial relationships that could be construed as a potential conflict of interest.
